# Three-Factor User Authentication and Key Agreement Using Elliptic Curve Cryptosystem in Wireless Sensor Networks

**DOI:** 10.3390/s16122123

**Published:** 2016-12-14

**Authors:** YoHan Park, YoungHo Park

**Affiliations:** School of Electronics Engineering, Kyungpook National University, Daegu 41566, Korea; hanny12@ee.knu.ac.kr

**Keywords:** user authentication, key agreement, biometric information, elliptic curve cryptosystem, wireless sensor networks

## Abstract

Secure communication is a significant issue in wireless sensor networks. User authentication and key agreement are essential for providing a secure system, especially in user-oriented mobile services. It is also necessary to protect the identity of each individual in wireless environments to avoid personal privacy concerns. Many authentication and key agreement schemes utilize a smart card in addition to a password to support security functionalities. However, these schemes often fail to provide security along with privacy. In 2015, Chang et al. analyzed the security vulnerabilities of previous schemes and presented the two-factor authentication scheme that provided user privacy by using dynamic identities. However, when we cryptanalyzed Chang et al.’s scheme, we found that it does not provide sufficient security for wireless sensor networks and fails to provide accurate password updates. This paper proposes a security-enhanced authentication and key agreement scheme to overcome these security weaknesses using biometric information and an elliptic curve cryptosystem. We analyze the security of the proposed scheme against various attacks and check its viability in the mobile environment.

## 1. Introduction

Wireless sensor networks (WSNs) are ad hoc networks composed of a number of sensor nodes with limited power, computation, storage and communication capabilities [1]. They provide effective solutions to a wide array of monitoring problems in various environments, such as battlefields, healthcare services and the smart grid [2]. Recently, sensor-attached things that communicate with neighboring things are enabling the development of the Internet of Things (IoT) environment [3]. For these reasons, WSNs have gained wide attention, in both the academic and industrial fields. However, the issue of securing and authenticating communication is problematic, because the nodes are vulnerable to attacks and do not have enough capacity for the secure storage of keys [4,5,6]. To solve these security issues, authentication and key agreement schemes using two-factor security, passwords and smart cards have attracted attention and have been studied widely in an effort to guarantee secure communication [7,8,9,10,11,12,13,14]. Unfortunately, many of them still suffer from various attacks and do not provide secure communication.

Several authentication and key agreement schemes for WSNs have been proposed. In 2010, Das [8] proposed a two-factor user authentication protocol for WSNs. He insisted the scheme withstood various attacks from users with the same identity, as well as from stolen-verifier attacks. However, He et al. [9], Khan and Alghathbar [10] and Chen and Shih [11] pointed out that Das’s scheme was vulnerable to insider and impersonation attacks, gateway node bypassing attacks and privileged-insider attacks and did not provide mutual authentication. Subsequently, each proposed their own authentication scheme to provide secure user authentication in WSNs. In 2012, Vaidya et al. [12] demonstrated that Das’s scheme [8], Khan and Alghathbar’s scheme [10] and Chen and Shih’s scheme [11] had security problems and that none of them provided key agreement. Vaidya et al. proposed a two-factor mutual user authentication scheme with key agreement for WSNs. In 2014, Kim et al. [13] presented that both gateway node bypassing attacks and user impersonation attacks were possible in Vaidya et al.’s scheme [12]. They proposed an authentication and key agreement scheme that resisted user impersonation and gateway node bypassing attacks. However, in 2015, Chang et al. [14] analyzed Kim et al.’s scheme [13] and found it had security vulnerabilities in the following areas: impersonation attacks, lost smart card attacks, man-in-the-middle attacks, violation of session key security and failure to protect user privacy. To solve these problems, Chang et al. [14] proposed a scheme that provided user privacy by using dynamic identities and provided better security functionality than Kim et al.’s scheme. However, we point out that Chang et al.’s scheme does not withstand several types of attacks and fails to provide a password update.

Recently, to improve the security of two-factor authentication schemes that are vulnerable to guessing attacks and subject to inefficient password change policies in WSNs, biometric-based user authentication schemes, combined with smart cards and passwords, have drawn considerable attention in research [15,16,17,18,19]. Biometric-based user authentication in the WSN becomes inherently more reliable and secure than traditional two-factor user authentication schemes [20]. Several advantages can be derived from the use of biometric keys over traditional passwords because they cannot be lost; they are unforgettable, difficult to copy, hard to forge and difficult to break. Therefore, biometric-based user authentication is considered to be more secure and reliable than conventional authentication schemes [20].

In this paper, we cryptanalyze Chang et al.’s scheme [14] and demonstrate the security weaknesses, such as password guessing attacks, lack of forward secrecy and inaccurate password updates. Further, we propose a biometric-based user authentication and key agreement scheme for WSNs using fuzzy extraction and an elliptic curve cryptosystem (ECC). The proposed scheme withstands security threats from malicious adversaries and insider users by using an ECC-based session key. Our scheme is also suitable for WSNs when compared to traditional authentication and key agreement schemes because it performs simple ECC operations, hash functions and exlusive OR (XOR) operations. We prove that our scheme provides mutual authentication using Burrows-Abadi-Needham (BAN) logic [21].

The remainder of this paper is organized as follows: In Section 2, we present our preliminary details, and Chang et al.’s scheme is reviewed in Section 3. In Section 4, we cryptanalyze Chang et al.’s scheme, and our proposed scheme is presented in Section 5. Finally, we analyze our proposed scheme in Section 6 and conclude with the findings of this work in Section 7.

## 2. Preliminaries

In this section, we introduce the notations used in this paper and then define the cryptographic system and primitives used as building blocks in our security system. Finally, we define security requirements for user authentication in WSNs.

### 2.1. Notations

The notations used throughout this paper are described in Table 1.

### 2.2. Elliptic Curves Cryptosystem

Let p,q be two large primes, and $E/F_{p}$ indicates an elliptic curve $y^{2}=x^{3}+ax+b$ over the finite field $F_{p}$. We denote by $G_{1}$ a *q*-order subgroup of the additive group of points of $E/F_{p}$. The discrete logarithm problem (DLP) is required to be hard in $G_{1}$. Mathematical problems in ECC are given as follows [22]:

**Definition** **1** (Elliptic curve discrete logarithm (ECDL) problem)**.** *Given a point element* $Q\in G_{1}$*, find an integer* $a\in Z_{p}^{*}$, such that Q=a×P*, where* a×P *indicates that the point P is added to itself for a times by the elliptic curve operation.*

**Definition** **2** (Elliptic curve computational Diffie–Hellman (ECDH) problem)**.** *For* $a,b\in Z_{p}^{*}$, given two point elements $a\times P,b\times P\in G_{1}$*, compute* $a\times b\times P\in G_{1}$.

**Definition** **3** (Elliptic curve decisional Diffie–Hellman (ECDDH) problem)**.** *For* $a,b,c\in Z_{p}^{*}$, given three point elements $a\times P,b\times P,c\times P\in G_{1}$*, decide whether* c×P=a×b×P *or not.*

We assume that the ECDDH problem is intractable, which may guarantee that there is no probabilistic polynomial time (PPT) algorithm to solve ECDDHP, ECCDHP and ECDDLP with non-negligible probability.

### 2.3. Fuzzy Extraction

We briefly describe the extraction process of key data from the given biometrics of a user using a fuzzy extractor. The output of a conventional hash function is sensitive, and it may also return completely different outputs even if there is little variation in the inputs. Note that the biometric information is prone to various noises during data acquisition, and the reproduction of the actual biometrics is hard in common practice. To avoid such a problem, a fuzzy extractor method [23] is preferred, which can extract a uniformly-random string and public information from the biometric template with a given error tolerance. In the reproduction process, the fuzzy extractor recovers the original biometric key data for noisy biometrics using a helper string. The fuzzy extractor consists of Gen (generate) and Rep (reproduce).

- $\mathrm{Gen}\left(BIO_{i}\right)=\left(R_{i},P_{i}\right).$ This probabilistic algorithm takes a biometric template $BIO_{i}$ as an input and then outputs a biometric key $R_{i}$, which is a uniform and random string, and a helper string $P_{i}$. $R_{i}$ can be the same under the assistance of $P_{i}$ even if the biometric information changes slightly.
- $\mathrm{Rep}\left(BIO_{i}^{\prime },P_{i}\right)=\left(R_{i}\right).$ This deterministic algorithm takes noisy biometric information $BIO_{i}^{\prime }$ and a helper string $P_{i}$ as inputs, then reproduces the biometric key $R_{i}$. To reproduce the same $R_{i}$, the metric space distances between $BIO_{i}$ and $BIO_{i}^{\prime }$ have to meet the given verification threshold.

### 2.4. Network Model

- $U_{i}$: A user who receives a smart card from GWN and uses it to access multiple servers. After a successful authentication process with $S_{j}$, the user is given access to mobile services. Furthermore, the user’s smart card is not tamper-resistant and can be lost or stolen by an adversary.
- $S_{j}$: A sensor node that collects information and provides services to users who successfully complete the authentication process. Sensors are not equipped with tamper-resistant hardware due to cost constraints, thus an adversary will know all of the keying materials stored in that sensor’s memory.
- GWN: A trusted third-party that generates system parameters. It provides smart cards to users and pre-shared keys to sensors. GWN is assumed to be trustworthy and never compromised by an adversary.

### 2.5. Security Requirements

According to recent studies [24,25], the user authentication scheme for WSNs should satisfy the following security requirements: (1) mutual authentication: the user $U_{i}$ and the sensor node $S_{j}$ should authenticate each other with the help of the gateway node GWN; (2) anonymity: any adversary A should not be able to obtain the real identity of the user $U_{i}$; (3) session key generation: after executing the authentication and key agreement phase, the user $U_{i}$ and the sensor node $S_{j}$ should generate a session key; (4) unconstrained by GWN: the GWN should not have or be able to compute the registered user’s information, such as the password and biometric template; (5) attack resistance: the scheme should withstand various attacks, such as off-line identity/password guessing, impersonation, smart card loss, man-in-the-middle and reply attacks; (6) efficient password update: it is required to change or update the users’ password without the participation of GWN.

## 3. Review of Chang et al.’s Authentication and Key Agreement Scheme

In this section, we review Chang et al.’s authenticated key agreement scheme. It comprises four phases: registration, login, authentication and key agreement, as well as password change.

### 3.1. Registration Phase

Step 1: $U_{i}$ chooses $ID_{i},pw_{i}$ and a random number $RN_{r}$, then computes $HPW_{i}=h(pw_{i}||RN_{r})$ and sends $\left\{ID_{i},HPW_{i}\right\}$ to GWN via a secure channel.
Step 2: GWN computes $HID_{i}=h(ID_{i}\left||K\right)$, $X_{S_{i}}=h(HID_{i}\left||K\right)$, $A_{i}=h(HPW_{i}\left|\right|X_{S_{i}})⊕HID_{i}$, $B_{i}=h\left(HPW_{i}⊕X_{S_{i}}\right)$, $C_{i}=X_{S_{i}}⊕h(ID_{S}||HPW_{i})$. Then, GWN sends the smart card $SC_{i}=\left(ID_{S},h\left(\cdot \right),A_{i},B_{i},C_{i},TID_{i}\right)$ to $U_{i}$ via a secure channel. GWN stores $\left(TID_{i},TID_{i}^{\circ },HID_{i}\right)$ in its storage, where $TID_{i}=RN_{G}$, $RN_{G}$ is a nonce, and $TID_{i}^{\circ }=\prime \prime \prime \prime$, where $TID_{i}^{\circ }=\prime \prime \prime \prime$ means $TID_{i}^{\circ }$ contains nothing.
Step 3: $U_{i}$ computes $XPW_{i}=h\left(pw_{i}\right)⊕RN_{r}$ and inserts it into $SC_{i}$.

### 3.2. Login Phase

Step 1: $U_{i}$ inputs $ID_{i}^{*}$ and $pw_{i}^{*}$ into $SC_{i}$.
Step 2: $SC_{i}$ computes $RN_{r}^{*}=h\left(pw_{i}^{*}\right)⊕XPW_{i}$, $HPW_{i}^{*}=h(pw_{i}^{*}||RN_{r}^{*})$, $X_{S_{i}}^{*}=C_{i}⊕h(ID_{S}||HPW_{i}^{*})$, $B_{i}^{*}=h(HPW_{i}^{*}⊕X_{S_{i}}^{*}$. Then, $SC_{i}$ verifies $B_{i}^{*}\overset{?}{=}B_{i}$. If it is valid, $SC_{i}$ computes $k_{i}=h(X_{S_{i}}^{*}\left|\right|T_{i})$, $DID_{i}=h(HPW_{i}^{*}\left|\right|X_{S_{i}}^{*})⊕k_{i}$, $M_{U_{i},G}=h(A_{i}\left|\right|X_{S_{i}}^{*}\left|\right|T_{i})$, where $T_{i}$ is the timestamp.
Step 3: $U_{i}$ sends $\left\{DID_{i},M_{U_{i},G},T_{i},TID_{i}\right\}$ to GWN.

### 3.3. Authentication and Key Agreement Phase

Step 1: GWN checks the validity of $T_{i}$ and retrieves $HID_{i}$ from $TID_{i}$. Then, GWN computes $X_{S_{i}}=h(HID_{i}\left||K\right)$, $k_{i}=h(X_{S_{i}}\left|\right|T_{i})$, $X^{*}=DID_{i}⊕k_{i}$, $M_{U_{i},G}^{*}=h(\left(X^{*}⊕HID_{i}\right)\left|\right|X_{S_{i}}\left|\right|T_{i})$, then checks $M_{U_{i},G}^{*}\overset{?}{=}M_{U_{i},G}$. If it is correct, GWN computes $X_{S_{j}}=h(SID_{j}\left||K\right)$, $M_{G,S_{j}}=h(DID_{i}||SID_{j}\left|\right|X_{S_{j}}\left|\right|T_{G})$, then sends $\left\{DID_{i},M_{G,S_{j}},T_{G}\right\}$ to $S_{j}$, where $T_{G}$ is the timestamp.
Step 2: $S_{j}$ checks the validity of $T_{G}$ and computes $M_{G,S_{j}}^{*}=h(DID_{i}||SID_{j}\left|\right|X_{S_{j}}^{*}\left|\right|T_{G})$, then checks $M_{G,S_{j}}^{*}\overset{?}{=}M_{G,S_{j}}$. If it is successful, $S_{j}$ computes $k_{j}=h(X_{S_{j}}\left|\right|T_{j})$, $Z_{i}=M_{G,S_{j}}^{*}⊕k_{j}$, $K_{S}=f\left(DID_{i},k_{j}\right)$, $M_{S_{j},G}=h(Z_{i}\left|\right|X_{S_{j}}^{*}\left|\right|T_{j})$, then sends $\left\{M_{S_{j},G},T_{j}\right\}$ to GWN, where $T_{j}$ is the timestamp.
Step 3: GWN checks the validity of $T_{j}$ and computes $k_{j}=h(X_{S_{j}}\left|\right|T_{j})$, $Z_{i}^{*}=M_{G,S_{j}}^{*}⊕k_{j}$, $M_{S_{j},G}^{*}=h(Z_{i}\left|\right|X_{S_{j}}^{*}\left|\right|T_{j})$, then checks $M_{S_{j},G}^{*}\overset{?}{=}M_{S_{j},G}$. If it is correct, GWN computes $M_{G,U_{i}}=h(DID_{i}\left|\right|M_{U_{i},G}^{*}\left|\right|k_{j}\left|\right|X_{X_{i}}\left|\right|T_{G}^{\prime })$, $y_{i}=k_{j}⊕h\left(k_{i}\right)$, $TID_{i_{new}}=h(HID_{i}\left|\right|T_{i})$, then sends $\left\{y_{i},M_{G,U_{i}},T_{G}^{\prime }\right\}$, where $T_{G}^{\prime }$ is the timestamp. Additionally, GWN updates $\left(TID_{i},TID^{\circ }\right)$ as $\left(TID_{i_{new}},TID_{i}\right)$.
Step 4: $U_{i}$ checks the validity of $T_{G}^{\prime }$ and computes $k_{j}=y_{i}⊕h\left(k_{i}\right)$, $M_{G,U_{i}}^{*}=h(DID_{i}\left|\right|M_{U_{i},G}\left|\right|k_{j}\left|\right|X_{S_{i}}\left|\right|T_{G}^{\prime })$, then checks $M_{G,U_{i}}^{*}\overset{?}{=}M_{G,U_{i}}$ If it is correct, $U_{i}$ computes $K_{S}=f\left(DID_{i},k_{j}\right)$ and updates $TID_{i}$ as $h(HID_{i}||T_{i})$.

### 3.4. Password Change Phase

Step 1: $U_{i}$ inputs $\left\{ID_{i}^{*},pw_{i}^{*},pw_{ni}\right\}$ into $SC_{i}$, where $pw_{ni}$ is a new password.
Step 2: The smart card computes $RN_{r}^{*}=h\left(pw_{i}^{*}\right)⊕XPW_{i}$, $HPW_{i}^{*}=h(pw_{i}^{*}||RN_{r}^{*})$, $X_{S_{i}}^{*}=C_{i}⊕h(ID_{s}||HPW_{i}^{*})$, $B_{i}^{*}=h\left(HPW_{i}^{*}⊕X_{S_{i}}^{*}\right)$, then checks $B_{i}^{*}\overset{?}{=}B_{i}$. If it is correct, $SC_{i}$ computes updated values $HPW_{ni}=h(pw_{ni}||RN_{r}^{*})$, $A_{ni}=A_{i}⊕h(HPW_{i}^{*}\left|\right|X_{S_{i}}^{*})⊕h(HPW_{ni}\left|\right|X_{S_{i}}^{*})$, $B_{ni}=h\left(HPW_{ni}⊕X_{S_{i}}^{*}\right)$, $C_{ni}=X_{S_{i}}^{*}⊕h(ID_{S}\left|\right|HPW_{ni}$. Then, $SC_{i}$ replaces $\left(A_{i},B_{i},C_{i}\right)$ with $\left(A_{ni},B_{ni},C_{ni}\right)$.

## 4. Security Weaknesses of Chang et al.’s Scheme

In this section, we analyze the security weaknesses of Chang et al.’s scheme [14]. Chang et al. cryptanalyzed Kim et al.’s scheme [13] and improved it by providing enhanced security properties. They claimed that their protocol could withstand various attacks. However, we show that their protocol is vulnerable to off-line password guessing attacks and does not provide perfect forward secrecy. We also show that their protocol cannot satisfy accurate password change. The capabilities of an adversary A [25] throughout this paper are as follows:

- An adversary A can be either a user or a sensor node, but not a gateway node [26].
- An adversary A has total control over the public communication channel. Thus, the adversary can intercept, insert, delete or modify any message transmitted via a public channel.
- An adversary A may steal a user’s smart card and extract the information stored in it by means of analyzing the power consumption [27].
- An adversary A can easily guess low-entropy passwords in an off-line manner, but the guessing of two secret parameters is computationally infeasible in polynomial time [28].

### 4.1. Off-Line Password Guessing Attack

Previous works [27] demonstrated that smart cards could be vulnerable to side channel attack, i.e., A could extract the information stored in the smart card $SC_{i}$. A chooses an arbitrary password $pw_{i}^{*}$, then computes to guess a correct password as follows:

$$\begin{array}{ccc} RN_{r}^{*} & = & XPW_{i}⊕h\left(pw_{i}^{*}\right)\\ HPW_{i}^{*} & = & h(pw_{i}^{*}||RN_{r}^{*})\\ X_{S_{i}}^{*} & = & C_{i}⊕h(ID_{S}||HPW_{i}^{*})\\ B_{i}^{*} & = & h(HPW_{i}^{*}⊕X_{S_{i}}^{*})\\ \;\mathrm{verifies}\;B_{i}^{*} & \overset{?}{=} & B_{i} \end{array}$$

If they are equal, A finds the correct password. Otherwise, A guesses another $pw_{i}^{*}$ and repeats the steps listed above until the correct password is found. In practical applications, people usually choose an easy-to-remember password for convenience, thus passwords could come from a very small dictionary. Therefore, A could find the correct password using a brute-force attack.

Even though Chang et al. has claimed that it is secure, once A guesses the password correctly, A can launch various attacks, such as impersonation, stolen verifier and lost smart card attacks. This is due to the fact that the scheme uses only a password to check the validity of users. Therefore, it is crucial to protect password guessing attacks and use various authentication factors to check the validity of users.

### 4.2. Lack of Perfect Forward Secrecy

In Chang et al.’s scheme, session key $K_{S}$ is computed as $h(DID_{i},k_{j})$. Once a long-term key of $S_{j}$, $X_{S_{j}}$, is disclosed to A, A can compute previous session keys as follows:

Step 1: A intercepts and stores all messages exchanged in previous sessions, such as $DID_{i}$ and $T_{i}$.
Step 2: A computes $k_{j}=h(X_{S_{j}}\left|\right|T_{j})$, then finally retrieves a previous session key $K_{S}=f\left(DID_{i},k_{j}\right)$.

This result indicates that Chang et al.’s scheme does not provide perfect forward secrecy. Furthermore, A who knows $X_{S_{j}}$ also can compute present and future session keys by intercepting messages via the public channel, indicating that Chang et al.’s scheme does not provide backward secrecy.

### 4.3. Incorrectness of Password Change

Chang et al.’s adopted Kim et al.’s password change phase; however, we found out that Kim et al.’s password update is not suitable for Chang et al.’s scheme. We demonstrate the incorrectness of the password change phase as follows:

Step 1: Once the user performs the password change phase, the previous password $pw_{i}$ is changed into $pw_{ni}$, and information in the smart card, $\left(A_{i},B_{i},C_{i}\right)$, is replaced with $\left(A_{ni},B_{ni},C_{ni}\right)$.
Step 2: Then, the user performs the login phase using the new password $pw_{ni}$; however, $U_{i}$ is not allowed to access for not computing the proper $RN_{r}$ from $XPW_{i}$. $XPW_{i}$ is not updated in the password change phase; therefore, $RN_{r}^{*}=XPW_{i}⊕h\left(pw_{ni}^{*}\right)\neq RN_{r}$ and, finally, $B_{i}^{*}\neq B_{i}$.

In addition, it is of no use to update the password if the password is revealed even one time because no other information, such as identity, is required to login and change the password. Therefore, regardless of whether a user changes the password, A can also change the password and be verified by the smart card.

## 5. The Proposed Three-Factor Authentication and Key Agreement Scheme

In this section, we propose a secure three-factor authentication and key agreement scheme for WSNs to overcome the security weaknesses in Chang et al.’s scheme. Based on Kim et al. and Chang et al.’s schemes, the proposed scheme provides better security functionality by using biometric information of the user and makes up for the password update inaccuracy. The proposed scheme consists of four phases: registration, login, authentication and key agreement and password change. The details of each phase are presented as follows.

### 5.1. Registration Phase

**Figure 1:**
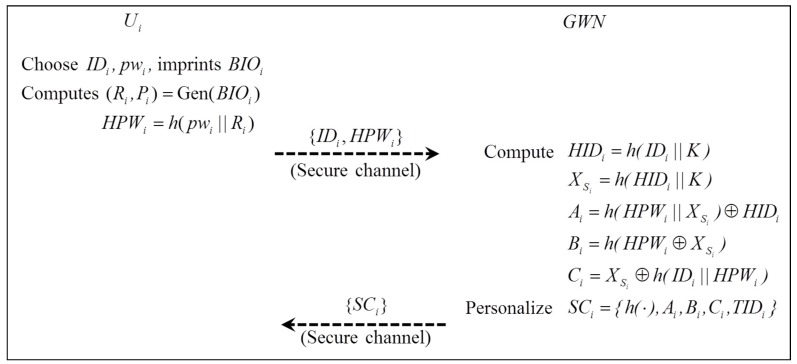
Registration phase.

A user $U_{i}$ registers the identity and password to GWN, then GWN generates a smart card $SC_{i}$ for $U_{i}$ and sends it to $U_{i}$ through a secure channel. Likewise, a sensor node $S_{j}$ is distributed with $\left(SID_{j},X_{S_{j}}\right)$, where $X_{S_{j}}=h(SID_{j}\left||K\right)$. Figure 1 illustrates the registration phase, which is performed as follows:

Step 1: $U_{i}\Rightarrow GWN$ : $\left\{ID_{i},HPW_{i}\right\}$
  $U_{i}$ chooses $ID_{i}$ and $pw_{i}$ and imprints $BIO_{i}$, then $U_{i}$ computes $\left(R_{i},P_{i}\right)=\mathrm{Gen}\left(BIO_{i}\right)$ and $HPW_{i}=h(pw_{i}\left|\right|R_{i})$ and sends $\left\{ID_{i},HPW_{i}\right\}$ to GWN through a secure channel.
Step 2: $GWN\Rightarrow U_{i}$ : $SC_{i}=\left\{h\left(\cdot \right),A_{i},B_{i},C_{i},TID_{i}\right\}$
  GWN computes $HID_{i}=h(ID_{i}\left||K\right)$, $X_{S_{i}}=h(HID_{i}\left||K)\right)$, $A_{i}=h(HPW_{i}\left|\right|X_{S_{i}})⊕HID_{i}$, $B_{i}=h\left(HPW_{i}⊕X_{S_{i}}\right)$, $C_{i}=X_{S_{i}}⊕h(ID_{i}||HPW_{i})$.
Step 3: GWN stores parameters $\left(TID_{i},TID_{i}^{\circ },HID_{i}\right)$, where $TID_{i}=RN_{G}$ ($RN_{G}$ is a nonce); $TID_{i}^{\circ }=\prime \prime \prime \prime$. $TID_{i}^{\circ }$ is empty at first time because $TID_{i}$ has not been updated; however, this parameter is required to check the correctness of the received $TID_{i}$ and retrieve $HID_{i}$ safely when GWN does not find a proper updated $TID_{i}$ in the case of an unsuccessful update process.
  Then, GWN issues the smart card $SC_{i}=\left\{h\left(\cdot \right),A_{i},B_{i},C_{i},TID_{i}\right\}$ and sends it to $U_{i}$ through a secure channel.

### 5.2. Login Phase

**Figure 2:**
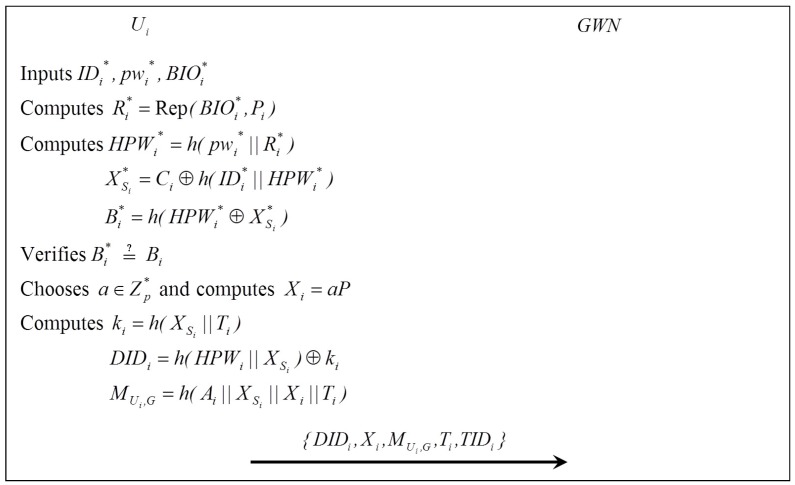
Login phase.

When $U_{i}$ tries to access the $S_{j}$, the login request is launched at first by $U_{i}$ with $SC_{i}$. Figure 2 illustrates the login phase, which is performed as follows:

Step 1: $U_{i}$ inserts $SC_{i}$, inputs $ID_{i}^{*}$, $pw_{i}^{*}$ and imprints $BIO_{i}^{*}$.
Step 2: $SC_{i}$ computes $R_{i}^{*}=\mathrm{Rep}\left(BIO_{i}^{*},P_{i}\right)$, $HPW_{i}^{*}=h(pw_{i}^{*}\left|\right|R_{i}^{*})$, $X_{S_{i}}^{*}=C_{i}⊕h(ID_{i}^{*}||HPW_{i}^{*})$, $B_{i}^{*}=h\left(HPW_{i}^{*}⊕X_{S_{i}}^{*}\right)$. Then, $SC_{i}$ verifies $B_{i}^{*}\overset{?}{=}B_{i}$. If it is correct, $SC_{i}$ generates a random number $a\in Z_{p}^{*}$ and computes $X_{i}=aP$, $k_{i}=h(X_{S_{i}}^{*}\left|\right|T_{i})$, $DID_{i}=h(HPW_{i}^{*}\left|\right|X_{S_{i}}^{*})⊕k_{i}$, $M_{U_{i},G}=h(A_{i}\left|\right|X_{S_{i}}^{*}\left|\right|X_{i}\left|\right|T_{i})$, where $T_{i}$ is the current timestamp.
Step 3: $U_{i}$ sends the login request message $\left\{DID_{i},X_{i},M_{U_{i},G},T_{i},TID_{i}\right\}$ to GWN.

### 5.3. Authentication and Key Agreement Phase

**Figure 3:**
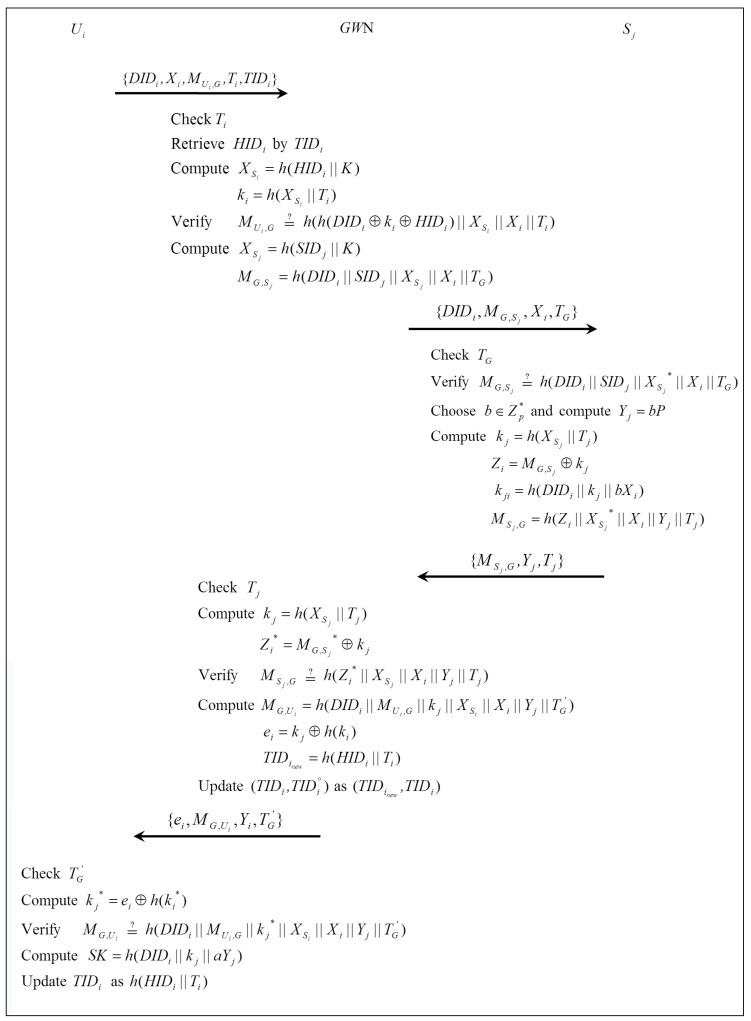
Authentication and key agreement phase.

In this phase, $U_{i}$ and $S_{j}$ authenticate each other and generate a common session key SK by the help of GWN. The trusted party GWN is interconnected with $U_{i}$ and $S_{j}$, respectively, and helps to establish a session key between $U_{i}$ and $S_{j}$; however, GWN is not able to derive the session key. Figure 3 illustrates the authentication and key agreement phase, which is performed as follows:

Step 1: $GWN\Rightarrow S_{j}$ : $\left\{DID_{i},X_{i},M_{G,S_{j}},T_{G}\right\}$
  After receiving $\left\{DID_{i},X_{i},M_{U_{i},G},T_{i},TID_{i}\right\}$, GWN checks the validity of $T_{i}$ and retrieves $HID_{i}$ from $TID_{i}$. If no $TID_{i}$ is found, GWN checks $TID_{i}^{\circ }$. If it still is not found, GWN rejects the login request; otherwise, GWN computes $X_{S_{i}}=h(HID_{i}\left||K\right)$ and $k_{i}=h(X_{S_{i}}\left|\right|T_{i})$. Then, GWN verifies $M_{U_{i},G}\overset{?}{=}h(\left(DID_{i}⊕k_{i}⊕HID_{i}\right)\left|\right|X_{S_{i}}\left|\right|X_{i}\left|\right|T_{i})$. If it is valid, GWN authenticates $U_{i}$ and computes $M_{G,S_{j}}=h(DID_{i}||SID_{j}\left|\right|X_{S_{j}}\left|\right|X_{i}\left|\right|T_{G})$, then sends $\left\{DID_{i},X_{i},M_{G,S_{j}},T_{G}\right\}$ to $S_{j}$, where $T_{G}$ is the current timestamp.
Step 2: $S_{j}\Rightarrow GWN$ : $\left\{M_{S_{j},G},Y_{j},T_{j}\right\}$
  After receiving $\left\{DID_{i},X_{i},M_{G,S_{j}},T_{G}\right\}$, $S_{j}$ checks the validity of $T_{G}$ and verifies $M_{G,S_{j}}\overset{?}{=}h(DID_{i}\left|\right|X_{i}\left|\right|X_{S_{j}}^{*}\left|\right|T_{G})$ using its stored secret value $X_{S_{j}}^{*}=h(SID_{j}\left||K\right)$. If it is valid, $S_{j}$ authenticates GWN and computes $k_{j}=h(X_{S_{j}}^{*}\left|\right|T_{j})$, $Z_{i}=M_{G,S_{j}}⊕k_{j}$, where $T_{j}$ is the current timestamp. Then, $S_{j}$ generates a random number $b\in Z_{p}^{*}$ and computes $Y_{j}=bP$ and a session key $SK=k_{ji}=h(DID_{i}\left|\right|k_{j}||bX_{i})$. Finally, $S_{j}$ computes $(M_{S_{j},G}=h(Z_{i}\left|\right|X_{S_{j}}^{*}\left|\right|X_{i}\left|\right|Y_{j}\left|\right|T_{j}))$ and sends $\left\{M_{S_{j},G},Y_{j},T_{j}\right\}$ to GWN.
Step 3: $GWN\Rightarrow U_{i}$ : $\left\{e_{i},M_{G,U_{i}},Y_{i},T_{G}^{\prime }\right\}$
  After receiving $\left\{M_{S_{j},G},Y_{i},T_{j}\right\}$, GWN checks the validity of $T_{j}$, computes $k_{j}=h(X_{S_{j}}\left|\right|T_{j})$, $Z_{i}^{*}=M_{G,S_{j}}^{*}⊕k_{j}$ and verifies $M_{S_{j},G}\overset{?}{=}h(Z_{i}^{*}\left|\right|X_{S_{j}}\left|\right|X_{i}\left|\right|Y_{j}\left|\right|T_{j})$. If it is valid, GWN authenticates $S_{j}$ and computes $e_{i}=k_{j}⊕h\left(k_{i}\right)$, $(M_{G,U_{i}}=h(DID_{i}\left|\right|M_{U_{i},G}\left|\right|k_{j}\left|\right|X_{S_{i}}\left|\right|X_{i}\left|\right|Y_{j}\left|\right|T_{G}^{\prime }))$, $TID_{i_{new}}=h(HID_{i}\left|\right|T_{i})$, where $T_{G}^{\prime }$ is the current timestamp. Then, GWN sends $\left\{e_{i},M_{G,U_{i}},Y_{i},T_{G}^{\prime }\right\}$ to $U_{i}$ and updates $\left(TID_{i},TID_{i}^{\circ }\right)$ as $\left(TID_{i_{new}},TID_{i}\right)$ in its storage.
Step 4: After receiving $\left\{e_{i},M_{G,U_{i}},Y_{i},T_{G}^{\prime }\right\}$, $U_{i}$ checks the validity of $T_{G}^{\prime }$, computes $k_{j}^{*}=e_{i}⊕h\left(k_{i}^{*}\right)$ and verifies $M_{G,U_{i}}\overset{?}{=}h(DID_{i}\left|\right|M_{U_{i},G}\left|\right|k_{j}^{*}\left|\right|X_{S_{i}}\left|\right|X_{i}\left|\right|Y_{j}\left|\right|T_{G}^{\prime })$. If it is valid, $U_{i}$ computes the session key $SK=k_{ij}=h(DID_{i}\left|\right|k_{j}||aY_{i})$. Finally, $U_{i}$ updates $TID_{i}$ as $h(HID_{i}||T_{i})$.

### 5.4. Password Change Phase

**Figure 4:**
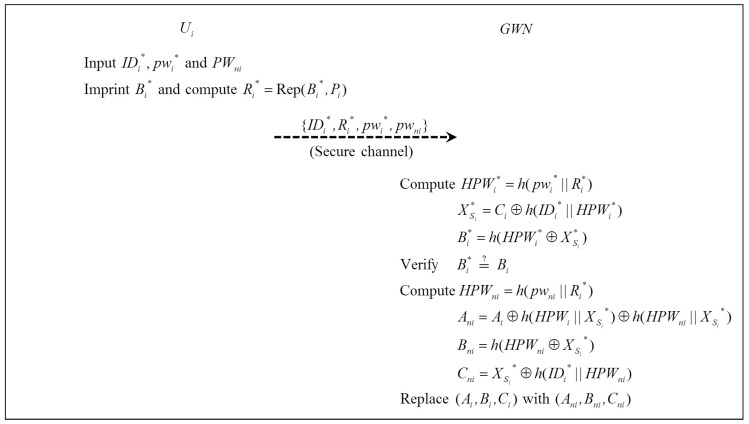
Password change phase.

When $U_{i}$ wants to change $pw_{i}$ with the new $pw_{ni}$, $U_{i}$ performs the password change phase. Figure 4 illustrates the password change phase, which is performed as follows:

Step 1: $U_{i}$ imprints $BIO_{i}^{*}$ and computes $R_{i}^{*}=\mathrm{Rep}\left(BIO_{i}^{*},P_{i}\right)$, then inputs $\left\{ID_{i}^{*},R_{i}^{*},pw_{i}^{*},pw_{ni}\right\}$ into $SC_{i}$.
Step 2: $SC_{i}$ computes $HPW_{i}^{*}=h(pw_{i}^{*}\left|\right|R_{i}^{*})$, $X_{S_{i}}^{*}=C_{i}⊕h(ID_{i}^{*}||HPW_{i}^{*})$, $B_{i}^{*}=h\left(HPW_{i}^{*}⊕X_{S_{i}}^{*}\right)$. Then, $SC_{i}$ verifies $B_{i}^{*}=B_{i}$ to check the validity of $U_{i}$. If it is correct, $SC_{i}$ computes updated values $HPW_{ni}=h(pw_{ni}\left|\right|R_{i}^{*})$, $A_{ni}=A_{i}⊕h(HPW_{i}\left|\right|X_{S_{i}}^{*})⊕h(HPW_{ni}\left|\right|X_{S_{i}}^{*})$, $B_{ni}=h\left(HPW_{ni}⊕X_{S_{i}}^{*}\right)$, $C_{ni}=X_{S_{i}}^{*}⊕h(ID_{i}^{*}||HPW_{ni})$. Then, $SC_{i}$ replaces $\left(A_{i},B_{i},C_{i}\right)$ with $\left(A_{ni},B_{ni},C_{ni}\right)$.

## 6. Analysis

In this section, we describe an analysis of our proposed authentication and key agreement scheme with respect to security and efficiency. We assume that the capabilities of the adversary are the same as those from our cryptanalysis of Chang et al.’s scheme in Section 4. We first prove the security of our scheme with BAN logic [21], then analyze the proposed scheme based on the security requirements for WSNs.

### 6.1. Proof of Authentication and Key Agreement Based on BAN Logic

Recently, security analyses about authentication and key agreement schemes in WSNs have been conducted using the BAN logic, which is a method to prove the security of mutual authentication and a session key [25,29]. In this section, we analyze the security of our proposed authentication scheme with BAN logic [21]. Table 2 illustrates notations used in BAN logic.

1. The BAN logic postulates:
  (a) Message meaning rule:
    $$\frac{P\;\text{believes}\;Q\overset{K}{↔}P,P\;\text{sees}\;{\left\{X\right\}}_{K}}{P\;\text{believes}\;Q\;\text{said}\;X}$$
  (b) Nonce-verification rule:
    $$\frac{P\;\text{believes fresh}\;(X),P\;\text{believes}\;Q\;\text{said}\;X}{P\;\text{believes}\;Q\;\text{believes}\;X}$$
  (c) Jurisdiction rule:
    $$\frac{P\;\text{believes}\;Q\;\text{controls}\;X,P\;\text{believes}\;Q\;\text{believes}\;X}{P\;\text{believes}\;X}$$
  (d) Freshness-conjuncatenation rule:
    $$\frac{P\;\text{believes fresh}\;(X)}{P\;\text{believes fresh}\;(X,Y)}.$$
2. Security goals:
  The proposed scheme should satisfy the following goals:
  *g*_1_. $U_{i}|\equiv U_{i}\overset{SK}{↔}S_{j}$
  *g*_2_. $S_{j}|\equiv U_{i}\overset{SK}{↔}S_{j}$
  *g*_3_. $U_{i}\left|\equiv S_{j}\right|\equiv U_{i}\overset{SK}{↔}S_{j}$
  *g*_4_. $S_{j}\left|\equiv U_{i}\right|\equiv U_{i}\overset{SK}{↔}S_{j}$
3. Idealized scheme:
  We transform our scheme into the idealized form as follows:
  *Msg*_1_. $U_{i}\to GWN:{\left(DID_{i},K,X_{i},T_{i}\right)}_{HID_{i}}$
  *Msg*_2_. $GWN\to S_{j}:{\left(DID_{i},SID_{j},K,X_{i},T_{G}\right)}_{X_{S_{j}}}$
  *Msg*_3_. $S_{j}\to GWN:{\left(DID_{i},SID_{j},K,X_{i},Y_{i},T_{j}\right)}_{X_{S_{j}}}$
  *Msg*_4_. $GWN\to U_{i}:{\left(DID_{i},k_{j},K,X_{i},Y_{i},T_{G}^{\prime }\right)}_{HID_{i}}$
4. Initiative premises:
  We make the assumptions about the initial state of the scheme to analyze the proposed scheme as follows.
  *p*_1_. $GWN|\equiv \#(T_{i})$
  *p*_2_. $GWN|\equiv \#(T_{j})$
  *p*_3_. $S_{j}|\equiv \#\left(T_{G}\right)$
  *p*_4_. $U_{i}|\equiv \#\left(T_{G}^{\prime }\right)$
  *p*_5_. $GWN|\equiv GWN\overset{X_{S_{j}}}{↔}S_{j}$
  *p*_6_. $S_{j}|\equiv GWN\overset{X_{S_{j}}}{↔}S_{j}$
  *p*_7_. $U_{i}|\equiv U_{i}\overset{HID_{i}}{↔}GWN$
  *p*_8_. $GWN|\equiv U_{i}\overset{HID_{i}}{↔}GWN$
  *p*_9_. $U_{i}|\equiv S_{j}\Rightarrow U_{i}\overset{SK}{↔}S_{j}$
  *p*_10_. $S_{j}|\equiv U_{i}\Rightarrow U_{i}\overset{SK}{↔}S_{j}$
    (The meanings of $p_{9}$ and $p_{10}$ are different from $g_{3}$ and $g_{4}$. $p_{9}$ and $p_{10}$ are not the goals that we want to deduce. These are widely-used premises as done in [29,30,31,32].)
5. Security analysis of the idealized form of the proposed scheme:
  *a*_1_. According to $Msg_{1}$, we could get:
    $$\begin{array}{c} s_{1}:GWN⊲{\left(DID_{i},K,X_{i},T_{i}\right)}_{HID_{i}} \end{array}$$
  *a*_2_. According to $p_{8}$, we apply the message-meaning rule to obtain:
    $$\begin{array}{c} s_{2}:GWN\left|\equiv U_{i}\right|∼{\left(DID_{i},K,X_{i},T_{i}\right)}_{HID_{i}} \end{array}$$
  *a*_3_. According to $p_{1}$, we apply the freshness-conjuncatenation rule to obtain:
    $$\begin{array}{c} s_{3}:GWN|\equiv \#{\left(DID_{i},K,X_{i},T_{i}\right)}_{HID_{i}} \end{array}$$
    Then, from $s_{2}$ and $s_{3}$, we apply the nonce-verification rule to obtain:
    $$\begin{array}{c} s_{4}:GWN\left|\equiv U_{i}\right|\equiv {\left(DID_{i},K,X_{i},T_{i}\right)}_{HID_{i}} \end{array}$$
  *a*_4_. According to $Msg_{2}$, we could get:
    $$\begin{array}{c} s_{5}:S_{j}⊲{\left(DID_{i},SID_{j},K,X_{i},T_{G}\right)}_{X_{S_{j}}} \end{array}$$
  *a*_5_. According to $p_{6}$, we apply the message-meaning rule to obtain:
    $$\begin{array}{c} s_{6}:S_{j}\left|\equiv GWN\right|∼{\left(DID_{i},SID_{j},K,X_{i},T_{G}\right)}_{X_{S_{j}}} \end{array}$$
  *a*_6_. According to $p_{3}$, we apply the the freshness-conjuncatenation rule to obtain:
    $$\begin{array}{c} s_{7}:S_{j}|\equiv \#{\left(DID_{i},SID_{j},K,X_{i},T_{G}\right)}_{X_{S_{j}}} \end{array}$$
    Then, from $s_{6}$ and $s_{7}$, we apply the nonce-verification rule to obtain:
    $$\begin{array}{c} s_{8}:S_{j}\left|\equiv GWN\right|\equiv {\left(DID_{i},SID_{j},K,X_{i},T_{G}\right)}_{X_{S_{j}}} \end{array}$$
  *a*_7_. According to $Msg_{3}$, we could get:
    $$\begin{array}{c} s_{9}:GWN⊲{\left(DID_{i},SID_{j},K,X_{i},Y_{i},T_{j}\right)}_{X_{S_{j}}} \end{array}$$
  *a*_8_. According to $p_{5}$, we apply the message-meaning rule to obtain:
    $$\begin{array}{c} s_{10}:GWN\left|\equiv S_{j}\right|∼{\left(DID_{i},SID_{j},K,X_{i},Y_{i},T_{j}\right)}_{X_{S_{j}}} \end{array}$$
  *a*_9_. According to $p_{2}$, we apply the the freshness-conjuncatenation rule to obtain:
    $$\begin{array}{c} s_{11}:GWN|\equiv \#{\left(DID_{i},SID_{j},K,X_{i},Y_{i},T_{j}\right)}_{X_{S_{j}}} \end{array}$$
    Then, from $s_{10}$ and $s_{11}$, we apply the nonce-verification rule to obtain:
    $$\begin{array}{c} s_{12}:GWN\left|\equiv U_{i}\right|\equiv {\left(DID_{i},SID_{j},K,X_{i},Y_{i},T_{j}\right)}_{X_{S_{j}}} \end{array}$$
  *a*_10_. According to $Msg_{4}$, we could get:
    $$\begin{array}{c} s_{13}:U_{i}⊲{\left(DID_{i},k_{j},K,X_{i},Y_{i},T_{G}^{\prime }\right)}_{HID_{i}} \end{array}$$
  *a*_11_. According to $p_{7}$, we apply the message-meaning rule to obtain:
    $$\begin{array}{c} s_{14}:U_{i}\left|\equiv GWN\right|∼{\left(DID_{i},k_{j},K,X_{i},Y_{i},T_{G}^{\prime }\right)}_{HID_{i}} \end{array}$$
  *a*_12_. According to $p_{4}$, we apply the the freshness-conjuncatenation rule to obtain:
    $$\begin{array}{c} s_{15}:U_{i}|\equiv \#{\left(DID_{i},k_{j},K,X_{i},Y_{i},T_{G}^{\prime }\right)}_{HID_{i}} \end{array}$$
    Then, from $s_{14}$ and $s_{15}$, we apply the nonce-verification rule to obtain:
    $$\begin{array}{c} s_{16}:U_{i}\left|\equiv GWN\right|\equiv {\left(DID_{i},k_{j},K,X_{i},Y_{i},T_{G}^{\prime }\right)}_{HID_{i}} \end{array}$$
  *a*_13_. Because $SK=h(DID_{i}||k_{j}||bX_{i})$, according to $s_{16}$ and $s_{12}$, we could produce:
    $$\begin{array}{c} s_{17}:U_{i}\left|\equiv S_{j}\right|\equiv U_{i}\overset{SK}{↔}S_{j}\;\left(\mathrm{Goal}3\right) \end{array}$$
    Likewise, $SK=h(DID_{i}||k_{j}||aY_{i})$, according to $s_{8}$ and $s_{4}$, we could produce:
    $$\begin{array}{c} s_{18}:S_{j}\left|\equiv U_{i}\right|\equiv U_{i}\overset{SK}{↔}S_{j}\;\left(\mathrm{Goal}4\right) \end{array}$$
  *a*_14_. According to $s_{17}$ and $p_{9}$, we apply the jurisdiction rule to produce:
    $$\begin{array}{c} s_{19}:U_{i}|\equiv U_{i}\overset{SK}{↔}S_{j}\;\left(\mathrm{Goal}1\right) \end{array}$$
    Likewise, according to $s_{18}$ and $p_{10}$, we apply the jurisdiction rule to produce:
    $$\begin{array}{c} s_{20}:S_{j}|\equiv U_{i}\overset{SK}{↔}S_{j}\;\left(\mathrm{Goal}2\right) \end{array}$$
  According to Goal 1, Goal 2, Goal 3 and Goal 4, we conclude that both $U_{i}$ and $S_{j}$ believe they share the session key.

### 6.2. Security Analysis against Various Attacks

• User anonymity and untraceability: Our scheme provides anonymity of users. The user $U_{i}$ does not reveal a real identity $ID_{i}$ in open channels; instead, GWN generates and sends a pseudonym identity $TID_{i}=HID_{i}=RN_{G}$ to $U_{i}$ in the registration phase and updates it as $TID_{i}=h(HID_{i}\left|\right|T_{i})$ before finalizing the session. The identity is dynamic for every session; thus, an adversary A cannot obtain the user’s true identity. The proposed scheme also provides untraceability by having all messages used in the session satisfy a freshness requirement. Therefore, A cannot trace the user.
• Perfect forward secrecy: A session key SK is computed as $h(DID_{i}||k_{j}||abP)$. Even though the long-term private keys $X_{S_{i}}$ and $X_{S_{j}}$ are disclosed to A, he/she cannot compute previous session keys, because it is hard to compute abP using $X_{i}$ and $Y_{i}$ due to the difficulty of ECDH. Thus, A cannot compute previous session keys using long-term private keys. Therefore, our scheme provides forward secrecy.
• Mutual authentication: In our scheme, $U_{i}$ and GWN authenticate each other, and GWN and $S_{j}$ authenticate each other, respectively. GWN authenticates $U_{i}$ by checking $M_{U_{i},G}\overset{?}{=}h(\left(DID_{i}⊕k_{i}⊕HID_{i}\right)\left|\right|X_{S_{i}}\left|\right|X_{i}\left|\right|T_{i})$. A needs to compute $X_{S_{i}}$ and $k_{i}$ to reconstruct $M_{U_{i},G}$; however, only a legal user can compute those values. $U_{i}$ authenticates GWN by checking $(M_{G,U_{i}}=h(DID_{i}\left|\right|M_{U_{i},G}\left|\right|k_{j}\left|\right|X_{S_{i}}\left|\right|X_{i}\left|\right|Y_{j}\left|\right|T_{G}^{\prime }))$. A needs to compute $k_{j}^{*}$ and $X_{S_{i}}$ to reconstruct $(M_{G,U_{i}}$; however, only a legal GWN can compute those values. Therefore, $U_{i}$ and GWN mutually authenticate. Similarly, $S_{j}$ authenticates GWN by checking $M_{G,S_{j}}$, and GWN authenticates $S_{j}$ by checking $M_{S_{j},G}$. Additionally, only legal $S_{j}$ and GWN can reconstruct them, then authenticate mutually. Therefore, our scheme provides proper mutual authentication.
• Off-line password guessing attack: A may attempt to guess the password $pw_{i}$ by extracting the values stored in the smart card $SC_{i}$. A could guess correctly if he/she generates a series of equations and computes the valid $B_{i}$ using guessing passwords. However, A is required to know the biometric information of the user, which cannot be forged, for generating equations. Therefore, it is infeasible to correctly guess the user’s password in our scheme.
• Smart card loss attack: A can extract values in the smart card by means of power analysis and other techniques. Suppose A obtains the user’s smart card and extracts stored parameters $\left\{h\left(\cdot \right),A_{i},B_{i},C_{i},TID_{i}\right\}$. From these values, A cannot obtain any useful information because the parameters are safeguarded with a one-way hash function, and $TID_{i}$ is just a nonce. Furthermore, A may attempt to log in by generating a login request message. However, A cannot even pass the login phase and generate a valid login request message without proper $ID_{i}$, $pw_{i}$ and $B_{i}$. Therefore, the proposed scheme withstands smart card loss attacks.
• User impersonation attack: A who somehow possesses a valid smart card $SC_{i}$ of $U_{i}$ and wants to access $S_{j}$ is required to generate and send a valid login request message $\left\{DID_{i},X_{i},M_{U_{i},G},T_{i},TID_{i}\right\}$ to GWN. A must know $HPW_{i}$ and $X_{S_{i}}$ to compute these values. However, in our scheme, $ID_{i},pw_{i}$ and $R_{i}$ are not revealed. Thus, A cannot compute the temporal key $k_{i}$ and generate a valid login request message. Therefore, our scheme is secure against the user impersonation attack.
• Man-in-the-middle attack and replay attack: A who knows public channel information and has the smart card $SC_{i}$ of $U_{i}$ may attempt to establish a secure channel with $S_{j}$. However, A cannot authenticate with GWN because A cannot generate a valid login request message, as mentioned above. In addition, those messages captured in a public channel are refreshed in every session, so that A cannot use them repeatedly. Therefore, our scheme withstands man-in-the-middle and replay attacks.
• Stolen verifier attack: A who obtains the verifier table of GWN may attempt to attack users to gain some advantages. However, A still cannot compute $HPW_{i}$, $X_{S_{i}}$ and $k_{i}$ and will fail to pass the login phase. Of course, A will fail to compute a login request message without $pw_{i}$ and $R_{i}$. Therefore, even if A has the verifier table, our protocol withstands stolen verifier attacks.
• Known-key attack: A session key SK is computed as $h(DID_{i}||k_{j}||abP)$, and $DID_{i}$, $k_{j}$ and abP are independent in each session. Though A, who somehow possesses each value, attempts to generate other session keys, he/she will find that they cannot successfully derive valid session keys. Therefore, our proposed scheme withstands known-key attacks.

We compare the functionality features of the proposed scheme with related user authentication schemes for WSNs in Table 3. ∘ denotes that the scheme provides the property; × denotes that the scheme does not provide the property; Δ denotes that the scheme does not provide the property when off-line password guessing attacks succeed; − denotes that the scheme does not concern the property.

### 6.3. Performance Comparisons

In Table 4, we compare the computational cost with related schemes. $T_{h}$ denotes the computation time for the hash function; $T_{x}$ denotes the XOR operation; $T_{F}$ denotes the fuzzy extraction; $T_{E}$ denotes the ECC multiplication; $T_{enc}$ denotes the encryption/decryption. The computation cost of ours is a bit higher than [13,14] because of the usage of biometrics and ECC, but it is considered to be operationally viable in WSNs [15,18]. Additionally, our proposed scheme provides the enhanced security functionalities and is secure against various attacks.

## 7. Conclusions

To provide improved security functionality for mobile services in WSNs, several user authentication and key agreement schemes have been proposed in the last few years. However, most of them cannot provide secure authentication and are vulnerable to security attacks.

In this paper, we analyzed the security weaknesses of Chang et al.’s scheme and found that it is vulnerable to off-line password guessing attacks and does not provide forward secrecy and accurate password updates. To address the security problems, we proposed a biometric-based user authentication and key agreement scheme. The proposed scheme withstands the security attacks described above and provides better security functionality than previous schemes by using biometric information and ECC. In addition, we provided security and efficiency analyses, which demonstrated that the proposed protocol is more secure than the previous schemes and operationally viable in WSNs.

## Figures and Tables

**Table 1:** Notations.

Notation	Meaning
p,q	two large primes
$U_{i}$	user *i*
$S_{j}$	sensor node *j*
GWN	gateway node
$SC_{i}$	smart card of the user $U_{i}$
$ID_{i}/pw_{i}$	identity/password of $U_{i}$
$BIO_{i}$	biometric template of $U_{i}$
$TID_{i}$	temporal identity of $U_{i}$
$SID_{j}$	identity of $S_{j}$
$ID_{S}$	identity of $SC_{i}$
A	adversary
*K*	a master secret of GWN
$G_{1}$	cyclic group of order *q*
*P*	generator of $G_{1}$
$T_{i},T_{j},T_{G}$	timestamps
⨁	XOR operation
||	concatenate operation
h(·)	a secure one-way hash function

**Table 2:** BAN logic notations.

Notations	Meaning
P∣≡X	*P* believes *X*
P⊲X	*P* sees *X*
P∣∼X	*P* once said *X*
P⇒X	*P* has jurisdiction over *X*
#(X)	*X* is fresh
$P\overset{K}{↔}Q$	*P* and *Q* may use the shared key *K*
SK	The session key shared between two principals
${\langle X\rangle }_{Y}$	*X* combined with the formula *Y*
${\left(X\right)}_{K}$	*X* hashed under the key *K*
${\left\{X\right\}}_{K}$	*X* encrypted under the key *K*

**Table 3:** Comparisons of the functionality features. ECC, elliptic curve cryptosystem.

	Kim et al.’ Scheme [13]	Chang et al.’ Scheme [14]	Yoon and Yoo’s Scheme [15]	Choi et al.’ Scheme [18]	Proposed Scheme
Provides user anonymity	×	∘	×	×	∘
Provides user untraceability	×	Δ	×	×	∘
Provides forward secrecy	×	×	∘	∘	∘
Provides secure password update	∘	×	−	−	∘
Provides mutual authentication	∘	∘	∘	∘	∘
Resists off-line password guessing attack	×	×	−	−	∘
Resists user impersonation attack	×	Δ	∘	×	∘
Resists lost smart card attack	×	Δ	∘	∘	∘
Resists stolen verifier attack	×	Δ	−	−	∘
Resists man-in-the-middle attack	×	Δ	∘	∘	∘
Resists replay attack	∘	∘	∘	∘	∘
Resist biometric recognition error	−	−	×	∘	∘
Usage of biometrics	×	×	∘	∘	∘
Usage of ECC	×	×	∘	∘	∘

**Table 4:** Comparisons of the computation costs.

Scheme		Computation Cost		
		Registration	Login & Authentication	Total
Kim et al.’s [13]	User	$2T_{h}+T_{x}$	$9T_{h}+9T_{x}$	$11T_{h}+10T_{x}$
	GWN	$6T_{h}+3T_{x}$	$8T_{h}+8T_{x}$	$14T_{h}+11T_{x}$
	Sensor	0	$2T_{h}+2T_{x}$	$2T_{h}+2T_{x}$
Chang et al.’s [14]	User	$2T_{h}+T_{x}$	$9T_{h}+5T_{x}$	$11T_{h}+6T_{x}$
	GWN	$5T_{h}+3T_{x}$	$10T_{h}+4T_{x}$	$15T_{h}+7T_{x}$
	Sensor	0	$4T_{h}+T_{x}$	$4T_{h}+T_{x}$
Yoon and Yoo’s [15]	User	$T_{h}$	$3T_{h}+2T_{x}+2T_{E}$	$4T_{h}+2T_{x}+2T_{E}$
	GWN	$2T_{h}+2T_{x}$	$4T_{h}$	$6T_{h}+2T_{x}$
	Sensor	0	$3T_{h}+2T_{E}$	$3T_{h}+2T_{E}$
Choi et al.’s [18]	User	$T_{h}+T_{F}$	$10T_{h}+2T_{x}+T_{F}+T_{enc}+2T_{E}$	$11T_{h}+2T_{x}+2T_{F}+T_{enc}+2T_{E}$
	GWN	$3T_{h}+3T_{x}$	$10T_{h}+T_{x}+2T_{enc}$	$13T_{h}+4T_{x}+2T_{enc}$
	Sensor	0	$6T_{h}+T_{enc}+2T_{E}$	$6T_{h}+T_{enc}+2T_{E}$
Proposed	User	$T_{h}+T_{F}$	$9T_{h}+4T_{x}+T_{F}+2T_{E}$	$10T_{h}+4T_{x}+2T_{F}+2T_{E}$
	GWN	$5T_{h}+3T_{x}$	$11T_{h}+4T_{x}$	$16T_{h}+7T_{x}$
	Sensor	0	$4T_{h}+T_{x}+2T_{E}$	$4T_{h}+T_{x}+2T_{E}$
